# Supplementary figures and images for: Diagnosis of Taenia solium infections based on “mail order” RNA-sequencing of single tapeworm egg isolates from stool samples
Source: PLoS Negl Trop Dis. 2021 Dec 10;15(12):e0009787. doi: 10.1371/journal.pntd.0009787 (PMC8694474; doi:10.1371/journal.pntd.0009787)

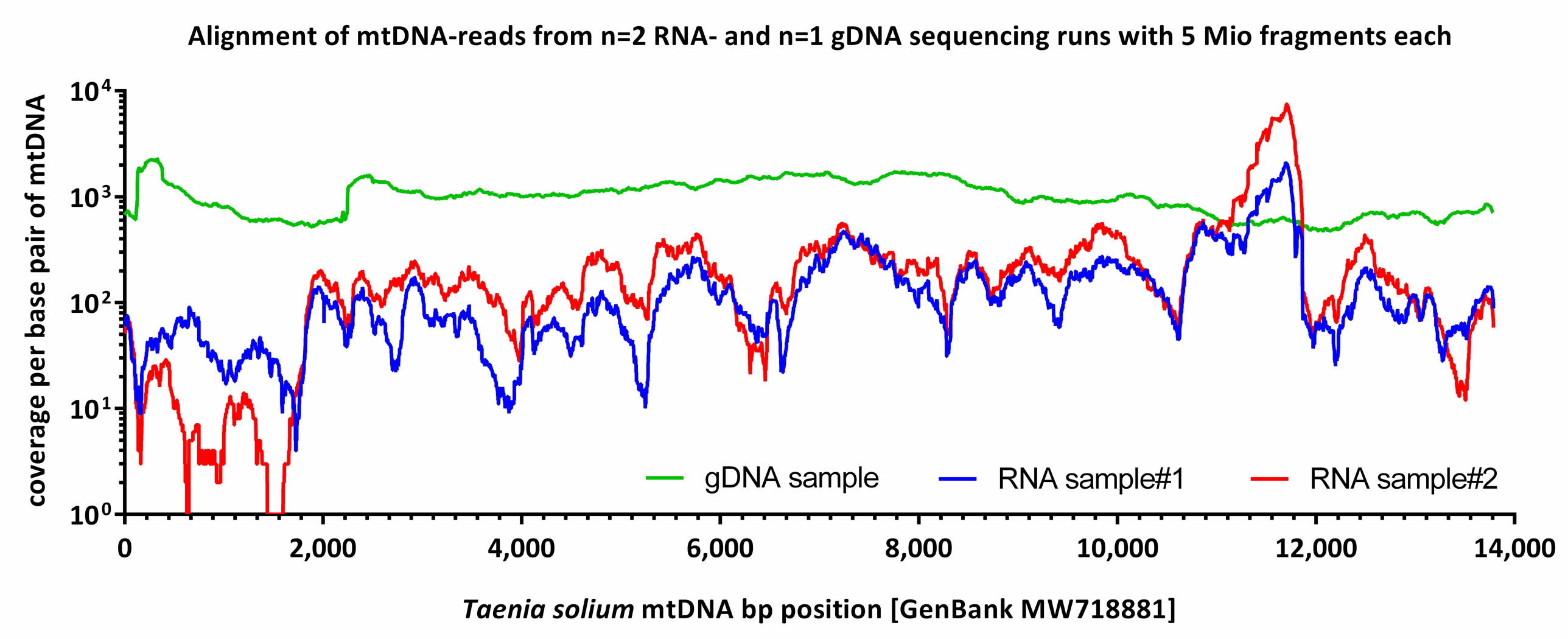

Supplement: S1 Fig — Please note the even distribution of coverage from the genomic fragments and the highly variable distribution on the mitochondrial RNA level. Nota bene, no inference can be made from this figure regarding the absolute amounts of mtDNA copies versus mtRNA transcripts, because RNA and DNA are not extracted from the same cell(s). For relative mapping quantities, please refer to the average coverage as well as the FPKM values on S2 Table). (TIF) [file pntd.0009787.s001.tif]

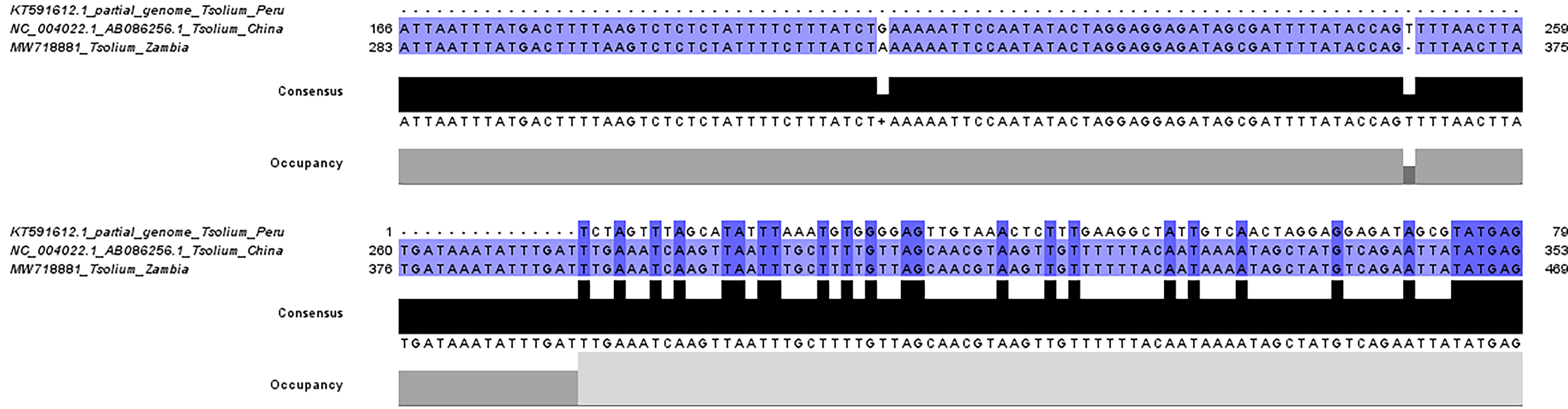

Supplement: S2 Fig — (TIF) [file pntd.0009787.s002.tif]

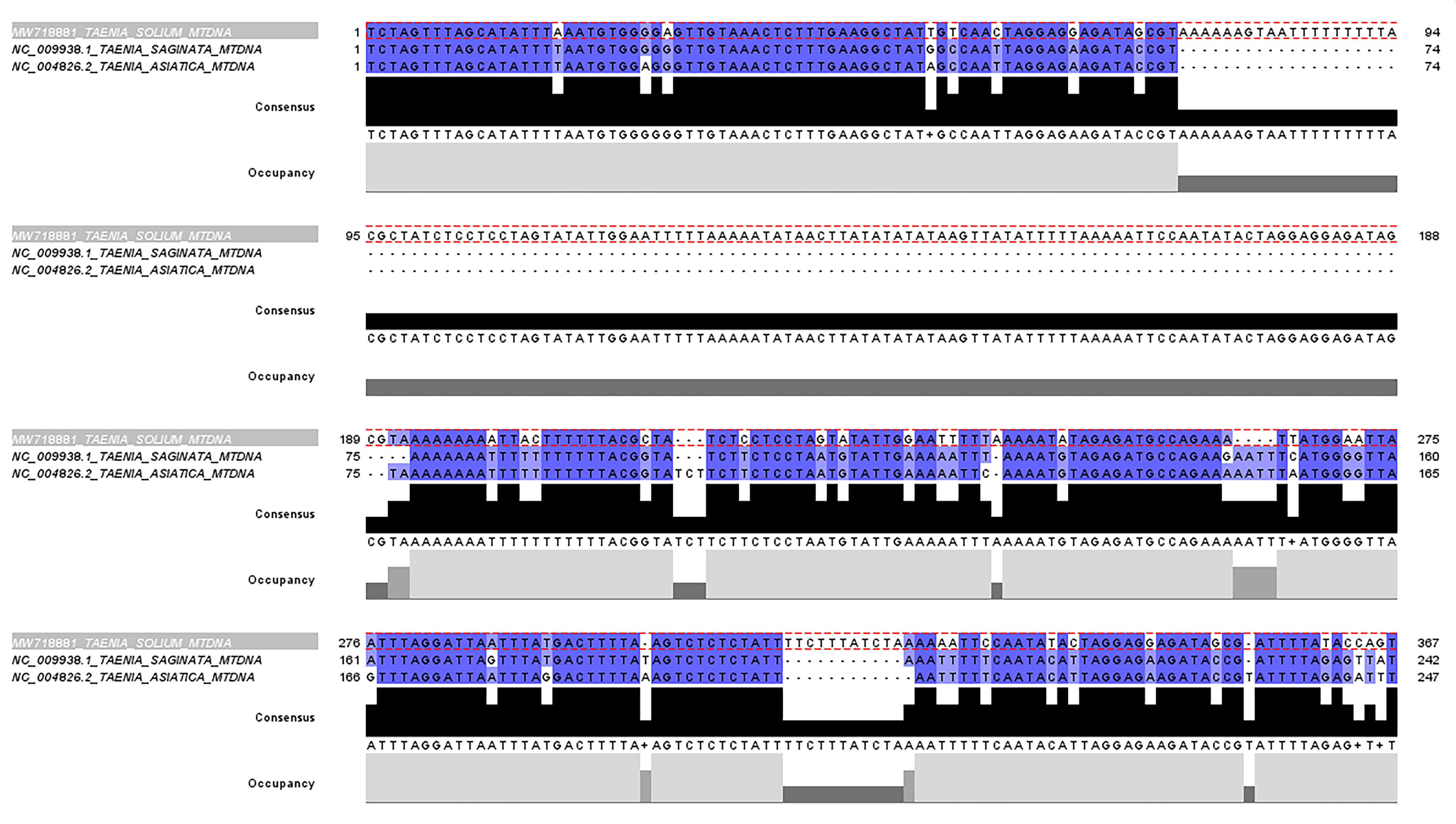

Supplement: S3 Fig — T. solium (MW718881), T. saginata (NC_009938.1), and T. asiatica (NC_004826.2). (TIF) [file pntd.0009787.s003.tif]
