# Supplementary material for: Diagnosis of Taenia solium infections based on “mail order” RNA-sequencing of single tapeworm egg isolates from stool samples
Source: PLoS Negl Trop Dis. 2021 Dec 10;15(12):e0009787. doi: 10.1371/journal.pntd.0009787 (PMC8694474; doi:10.1371/journal.pntd.0009787)
Supplement: Attachment — Submitted filename: 20211110_Response_to_reviewers.pdf [file pntd.0009787.s008.pdf]

CHARITÉ CAMPUS VIRCHOW-KLINIKUM

Otto Heubner Centrum für Kinder- und Jugendmedizin

CVK - Augustenburger Platz 1- 13353 Berlin

Prof. Xiao-Nong Zhou
Associate Editor
Prof. Hélène Carabin
Deputy Editor
PLoS Neglected Tropical Diseases

Klinik für Pädiatrie
mit Schwerpunkt Neurologie
Direktorin: Prof. Dr. med. Angela M. Kaindl

Prof. Dr. Markus Schuelke, MD
Telefon +49 30 450 566112
Telefax +49 30 450 7566920
Email markus.schuelke@charite.de

Berlin, November 8, 2021

Dear Editors,

the authors would like to submit a revised version of our manuscript entitled "**Diagnosis of *Taenia solium* infections based on "mail order" RNA-sequencing of single tapeworm egg isolates from stool samples**" to be considered for publication by the *PLoS Neglected Tropical Diseases* as an *Original Article*.

We very much thank the editors and reviewers for their positive and detailed comments. We have taken their comments into careful consideration while revising the manuscript and we provide a point-to-point answer below.

Comments to the editor

Figure files: We have run all our images through the PACE diagnostic tool and have now uploaded the analyzed and renamed images.

Data requirements: We have uploaded the raw data (FASTQ-files) and the processed sequencing data (*bam files of alignments, coverage files, FPKM abundancy files) into the GEO database (accession number GSE175668) and into the NCI RefSeq database (accession MW718881 for the *T. solium* mtDNA sequence).

Reproducibility: We have generated a laboratory protocol on protocols.io with a DOI (dx.doi.org/10.17504/protocols.io.bzx6p7re) that will be published, once the article is finally accepted. Meanwhile the link can only be accessed by people who exactly key in the above mentioned URL into an internet browser.

References: The reference list has been checked, complies to the PLOS NTD layout and does not contain retracted publications. We have added two references (#34 and #35) in order to reply to the comments of reviewer #2.

Comments to Reviewer #1

We thank the reviewer #1 for his/her positive feedback.

Minor edit:- Line 150: "'Here we present...'" (Please insert the word 'we' here).

Answer: The typo has been corrected

Comments to Reviewer #2

We also thank reviewer #2 for his/her encouraging remarks.

Line 192, "for up to five days", I want to know how long the samples can be stored at most?

Answer: ***We have added the following passage to the manuscript*:** "However, we did not perform a systematic time series to investigate how long the intact eggs could be stored in the field to obtain a good RNA sequencing result. The longest storage time was 8 days, which would be the maximum transport time from remote rural areas to a laboratory with access to international postal services. Considering that eggs in the wild can survive for several weeks or even months and remain infectious, i.e., "biologically intact" [3], we assume that much longer storage times would be possible, provided that Taenia spp. eggs remain intact."

Line 218, "Preparation of borosilicate needles for egg disruption", the process is a little complex, is there any substitute that can be purchased?

Answer: Special glass needles can be obtained from Fisher Scientific (E5242952008), but are expensive. Our protocol aims to use prefabricated consumables as little as possible. In our experience, on-site technical staff learned the technique in less than a day. In addition, the heating/melting process made the glass needles "naturally" RNAse-free, which is a prerequisite for isolating intact RNA for sequencing. Therefore, as mentioned in the manuscript, we recommend preparing the needles just before the intended egg breaking and protecting the glass tips from any skin contact.

We have now prepared a video illustrating the preparation, the pulling, breaking and sealing of the capillaries under the DOI (the video will be published after final acceptance) http://dx.doi.org/10.6084/m9.figshare.16955734

The reviewers will have access to this video already now by the following private link: https://figshare.com/s/412683e15c6eb7b36899.

Line 238, "8 Taenia eggs", why select 8? fewer than 8 is OK?

Answer: In an average stool specimen of an infected individual, we do find between 5-10 *Taenia spp.* eggs, so we took an "average" of 8. In our hands, we needed more *Taenia* eggs for the nested PCR on genomic DNA than for the "mail order" sequencing. The nested PCR on genomic DNA from only 1-2 eggs did not work.

Line 261, "Custom single-cell RNA-sequencing of the samples", as one of the main technologies of the developed workflow, I think the authors should pay more detailed descriptions of the custom single-cell RNA-sequencing of Taenia ssp. eggs.

Answer: We thank the reviewer for this comment. We have now presented the details of the process in some more detail in the manuscript, but we would also like to draw the reader's attention to a detailed protocol published on protocols.io at http://dx.doi.org/10.17504/protocols.io.bzx6p7re. The protocol describes the process point-by-point and we have added comments on crucial steps and caveats.

Modified text: "Upon arrival at the sequencing facility, sample quality was assessed using an Agilent 2100 Bioanalyzer and samples with sufficient RNA quality were used for library construction using the unstranded SMART-Seq 2 protocol [25]. Sequencing was done on the BGISEQ-500 platform [24]. We modified this protocol by employing a modified SMART-Seq buffer containing 18.5 mM dATP, 18.5 mM dCTP, 18.5 mM dGTP, 18.5 mM dTTP, 7.4 U/μL RNAse Inhibitor, and 0.7% Triton-X100 and by proceeding immediately to Whole Transcriptome Amplification without carrying out the lysis step published in the original SMART-Seq 2 protocol. The remaining steps of the library preparation are based on the SMART-Seq 2 protocol with the reverse transcription of poly(A)$^+$ RNA and template switching being carried out using oligo(dT) primers containing template-switching oligos (TSOs) and cDNA being amplified using PCR with indexed primers. Circularization and sequencing were performed based on the DNA nanoball technology proprietary to BGI.

Line 388, "average FPKM values", The use of FPKMs for calculating differential expression of genes across samples. This approach has been proven to be unacceptable for the purpose of differential expression analyses. See these references for clarification and alternative methods: "Misuse of RPKM or TPM normalization when comparing across samples and sequencing protocols" - https://rnajournal.cshlp.org/content/ early/2020/ 04/13/rna.074922. 120". A survey of best practices for RNA-seq data analysis" - https://www.ncbi. nlm.nih.gov/pmc/articles/ PMC4728800/

Answer: We thank the reviewers for this comment and the reference to the two publications, which we have studied thoroughly. However, to start with, we did not compare the expression levels of genes *between* two samples, but *within* the same sample, e.g. expression of mtDNA derived RNA *versus* nDNA derived RNA. The two samples are mere biological replicates. According to the publication of Zhao *et al.* (2020) we would be "allowed" to use FPKM values here because (**i**) we used the same RNA-isolation and enrichment protocol (SMART-Seq 2, non-stranded protocol, Illumina) with poly(A)$^+$-selection. (**ii**) the samples derived from the same kind organism (*Taenia solium*) and cell type (egg), (**iii**) we did not perform a comparison between samples but within the same sample (mtDNA *versus* nDNA encoded genes), (**iv**) for FPKM normalization with the StringTie algorithm we did not remove highly expressed genes, because it was the aim of this study to exactly identify those highly expressed genes and to provide a measure for their abundance in comparison to "standard" house-keeping genes.

We have added the following passage: "We are aware of the fact that FPKM values are not always well suited to compare mRNA expression levels between samples [32]. However, here we compared the expression levels between genes

of the same sample in two biological replicates from the same species and tissue that had been processed in parallel, regarding RNA-extraction, poly(A)+-selection, and NGS-runs. For FPKM normalization with the StringTie algorithm [33] we did not remove highly expressed genes, because it was the aim of this study to exactly identify those highly expressed genes and to provide a measure for their abundance in comparison to ”standard” house-keeping genes.”

We hope to have answered all the reviewers’ comments adequately and that you now consider our manuscript to be publishable in *PLoS Neglected Tropical Diseases.*

Yours sincerely,

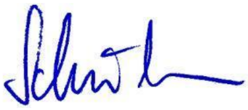

Markus Schuelke, MD
